# Cervical Cancer Screening by Refugee Category in a Refugee Health Primary Care Clinic in Calgary, Canada, 2011–2016

**DOI:** 10.1007/s10903-022-01345-5

**Published:** 2022-03-01

**Authors:** Molly Whalen-Browne, Rachel Talavlikar, Garielle Brown, Kerry McBrien, Mei-ling Wiedmeyer, Eric Norrie, Gabriel Fabreau

**Affiliations:** 1 Department of Family Medicine, Cumming School Medicine, University of Calgary, Calgary, AB Canada; 2 O’Brien Institute of Public Health, University of Calgary, Calgary, AB Canada; 3 Department of Medicine, Cumming School Medicine, University of Calgary, Calgary, AB Canada; 4 Department of Family Practice, University of British Columbia, Vancouver, BC Canada; 5 Mosaic Refugee Health Clinic, Calgary, AB Canada; 6 Department of Family Medicine, Faculty of Medicine & Dentistry, University of Alberta, Whyte Avenue PO, PO Box 95065, Edmonton, AB T6E 0E5 Canada

**Keywords:** Refugee health, Cervical cancer, Screening, Primary care

## Abstract

Newly arrived refugees and refugee claimants experience low cervical cancer screening (CCS) rates in Canada. We investigated CCS at a dedicated refugee clinic. We completed a retrospective cohort study among patients at the Mosaic Refugee Health Clinic in Calgary, Canada, between 2011 and 2016. We investigated CCS offers and completion by refugee category. We then used multivariable logistic regression to estimate the association of CCS screening and refugee category, accounting for sociodemographic and clinical factors. We included 812 refugees. Most were married (71%) and had limited English proficiency (57%). Overall, 88% and 77% of patients were offered and completed screening, respectively. Compared to government assisted refugees, privately sponsored refugees completed CCS more often (OR 1.60, 95% CI [1.02–2.49]). A dedicated refugee clinic may provide effective CCS to newly arrived refugees irrespective of refugee category, insurance status or other barriers.

## Background

### Cervical Cancer Screening and Barriers

Cervical cancer is a preventable disease, the fourth most commonly diagnosed cancer in women and the fourth most common cause of female cancer-related mortality worldwide [1]. Cervical cancer screening (CCS), including the Papanicolaou (Pap) smear, have greatly reduced the associated morbidity and mortality globally [2–4]. Despite this, several marginalized populations, including recently arrived refugees and refugee claimants (claimants), have below average CCS rates in Canada and the United States [5–10]. Refugees in high-income countries are of particular concern given that limited screening exists in many countries of origin, and refugees face additional barriers to accessing preventative healthcare services [11]. Factors that may influence CCS rates for refugees include: uncertainty for both patients and providers regarding their healthcare access and health insurance coverage, few structural and social supports, and refugees competing priorities navigating immigration systems [6, 8, 10].

Factors associated with lower CCS rates among refugees and immigrants include: recent arrival, visible minority status, limited English or French proficiency, not having a regular physician, low education and low-income status [10, 12–15]. Barriers to CCS in this population have also been linked to cultural beliefs and values around illness and screening, clinician characteristics such as being English speaking or male, as well as feelings of modesty and embarrassment around pelvic exams and sexual health [5, 9, 16–18].

### Refugee Categories and Clinical Care in Canada

Refugee claimants arrive in Canada and claim asylum either at their point of entry or after they are already in the country. Many wait multiple years for their cases to be heard by the Immigration Review Board with limited social and government supports, thus representing among Canada’s most vulnerable newcomers [19]. From 2012 to 2016, this situation worsened as restrictions to the Interim Federal Health Program (IFHP), Canada’s federally funded temporary health insurance program for refugees, resulted in claimants’ health coverage being limited to care related to perceived public health risks [20]. As a result, claimants’ healthcare access became much more difficult, including access to preventative care such as CCS. Other refugee categories including those sponsored and supported by family and private organizations [privately sponsored refugees (PSR)], and those supported and prioritized for resettlement by the Canadian government [government assisted refugees (GAR)], maintained full coverage. These changes were reversed in 2016, with full coverage restored for all refugee categories [21–23].

Over several decades, dedicated primary care-based refugee health clinics have emerged among refugee receiving countries such as Canada, the United States and Australia [24, 25]. As multidisciplinary clinics, they aim to reduce barriers, provide free or low-cost and comprehensive care with cultural humility, despite clinical complexity. However, while some studies recommend these dedicated clinics, limited evidence exists to show if these clinics do improve preventative care such as CCS for this vulnerable population, especially amidst major health policy changes such as the IFHP cuts [16, 26, 27].

### Objectives

To address these questions, we conducted a retrospective cohort study to determine how many eligible patients were offered, and subsequently received CCS, and whether this was impacted by refugee category, at a dedicated primary care refugee clinic in Canada during a period affected by IFHP cuts. We hypothesized a dedicated refugee clinic would provide high CCS completion rates, but despite access to specialized health care, claimants would experience lower rates of offered and completed CCS compared to other refugees.

## Methods

### Study Design and Participants

**Fig. 1:**
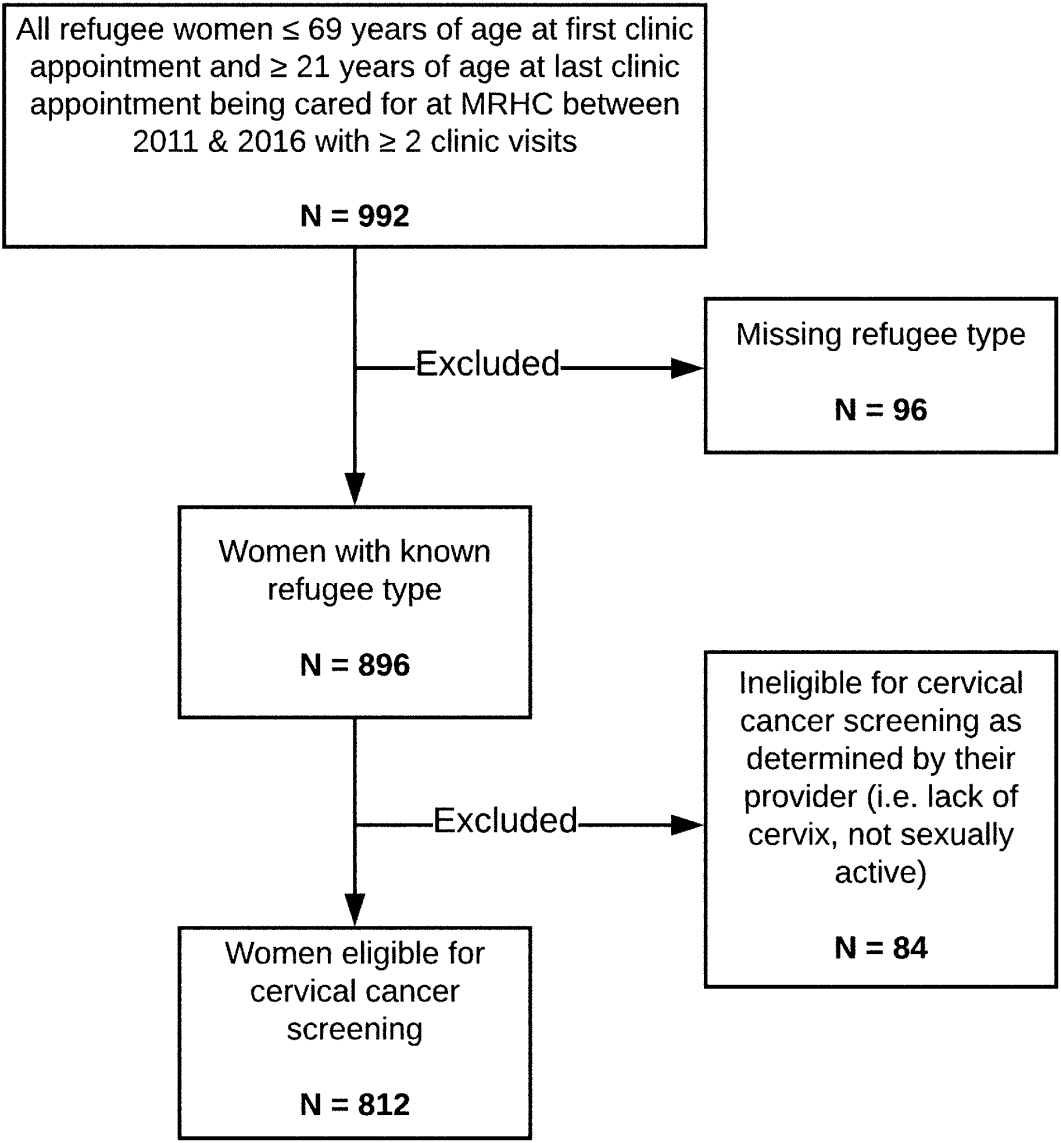
Study cohort inclusion criteria and flow chart at the Mosaic Refugee Health Clinic (MRHC), 2011–2016. *This age range allowed us to capture all women who were eligible for cervical cancer screening according to the age guidelines at some point during their time at the clinic

CCS guidelines in Alberta from 2011 to 2016 recommended screening every three years for sexually active females with a cervix ages 21–69, who were three years after sexual debut [28–30]. We therefore assembled a retrospective cohort of all refugee patients assigned female sex at birth, aged 21 or older at last clinic appointment and 69 or younger at first clinic appointment, who received any care at the Mosaic Refugee Health Clinic (MRHC) between January 1st, 2011 and December 31st, 2016. MRHC physicians routinely explain CCS via the Pap test to eligible patients during their initial intake visit, and offer and complete CCS during the second visit, to allow for improved patient comfort and rapport with the provider. As such, only patients deemed eligible to receive CCS by their provider (i.e., with a cervix and previously sexually active) and who had at least two appointments during the study period were included. An update to guidelines in May 2016 recommended starting CCS at age 25; however, we continued to include patients between ages 21–69 for the final months of the study period as we assumed a delayed uptake of the new guidelines (Fig. 1).

### Setting

The MRHC is a specialized interdisciplinary refugee clinic that cares for the majority of newly arrived refugees in Calgary, Canada—a metropolitan centre with approximately 1.3 million inhabitants—for up to two years after their arrival in Canada [25, 31]. It functions as a refugee medical home within a primary care network [32], but also provides some specialty care hosting a large multidisciplinary team that includes: family physicians, specialist physicians, chronic disease nurses, dieticians, pharmacists, psychologists, social workers, patient navigators and administrative staff. The clinic provides care to all its patients, irrespective of refugee category or health insurance status.

### Data Sources and Study Variables

Study data were obtained from electronic medical records (EMR) at the MRHC via a combination of manual and electronic extraction. We extracted CCS test results that were input directly into the EMR from the local clinical laboratory. We included the following study variables: refugee category, age at last appointment (in order to capture those who became eligible for CCS during their time at the clinic), United Nations global region of birth [33], patient identified marital status at intake, English proficiency (determined by clinicians at intake based on need for interpretation), medical complexity (determined by the number of individual diagnoses documented in the EMR at the time of chart review), length of time receiving care at the clinic, and CCS Pap test result where available. We defined the length of time a patient received care at MRHC as last appointment date minus intake date, converted to months. The number of clinical diagnoses each patient had were used to account for the impact medical complexity can have on preventative screening (i.e., more clinical complexity could detract from preventative care or increase the frequency of clinical visits and the probability of CCS testing).

Our primary exposure of interest was Canadian refugee category, defined as PSR, GAR, or claimant. Our primary outcome of interest was CCS (Pap test completion) and was determined by whether test results were present in the EMR. Our secondary outcome of interest was whether CCS screening test was offered. We assumed if the EMR contained a Pap test result then the patient had both completed (primary outcome) and had been offered (secondary outcome) a test. If multiple results were found, the first was used. If EMR Pap test results were not found, we searched clinician notes for explicit documentation regarding CCS having been offered. If clinical notes documented screening counselling and a test offer, we considered the test had been offered. If neither test result nor screening-specific clinical notes were found in the EMR, we assumed CCS had not been offered. We excluded patients who were missing refugee category (primary exposure) data. Finally, to investigate cervical cancer prevalence we performed an unstratified exploratory sub-group analysis among study subjects with available CCS pathology results.

### Bias and Age Bands

We used patient age at last appointment to include women who were aged 69 at intake but older at last appointment (maximum length of time in clinic 2 years). We expanded our age bands to capture all patients aged 21–69 years during our study period, which provided an age at last appointment range between 21 and 71 years.

We anticipated the relationship between age and our outcomes of interest would not be linear given the age ranges for CCS are specified in national and provincial clinical guidelines. We visually inspected outcome data by age and confirmed a non-linear relationship with large changes in outcomes observed below 25 and above 64 years of age, and similar frequencies between 25 and 65. Thus, we categorized patient’s ages at last appointment into three age bands: 21–24, 25–64 and 65–71 years respectively. The upper age band allowed us to capture all patients who were eligible for CCS at any point during their time at the clinic, even if they aged out of the guideline during that time.

### Statistical Methods

We compared patient characteristics among refugee categories (PSR, GAR, and claimant) using ANOVA, Kruskal–Wallis and Chi-square or Fisher’s exact tests for normally distributed, non-normally distributed, and categorical variables, respectively. We used Chi-square tests to compare the proportion of those in each refugee category who were offered and who completed CCS.

We used multivariable logistic regression models to estimate the association between refugee category (our primary exposure of interest), and the two outcomes of interest: CCS test completion (primary outcome) and CCS test offered (secondary outcome), while accounting for important sociodemographic and clinical factors which were included in the models as covariates [26, 27]. We calculated odds ratios for the outcomes of interest using GARs as the reference group, as they were the largest group (Table 1). In our models we accounted for age category (21–24, 25–64 and 65–71 years), United Nations global region of birth (Africa, Americas, Asia, Europe) [32], marital status (married/common law, divorced/separated, single, widowed, unknown), English proficiency (limited to none, moderate, fluent), total time in clinic (in months) and number of medical diagnoses. We considered a P value of < 0.05 significant for all tests. Statistical analysis was done using STATA 14 statistical software.

**Table 1:** Sociodemographic and clinical characteristics of patients in cohort by refugee category at the Mosaic Refugee Health Clinic, 2011–2016 (N = 812)

Characteristic	PSR^a^ n = 299	GAR^b^ n = 389	RC^c^ n = 124	P value
Age at last appointment (years)—median [IQR ^d^]	34 [29–41]	33 [28–42]	35 [29–46]	0.24
Age Bands^f^—n (%)				
21–24 years	29 (10)	49 (13)	11 (9)	
25–64 years	266 (89)	333 (86)	111 (90)	0.68
65–71 years	4 (1)	7 (2)	2 (2)	
Region of birth—n (%)				
Africa	220 (74)	141 (36)	38 (31)	< 0.01
Americas	1 (0)	9 (2)	29 (23)	
Asia	77 (26)	239 (61)	34 (27)	
Europe	1 (0)	0 (0)	23 (19)	
Marital status—n (%)				
Married/Common-law	195(65)	290 (75)	88 (71)	
Divorced/Separated	23 (8)	25 (6)	6 (5)	
Single	51 (17)	54 (14)	23 (19)	< 0.01
Widowed	28 (9)	17 (4)	4 (3)	
Unknown	2 (1)	3 (1)	3 (2)	
English proficiency—n (%)				
Limited to none	124 (42)	281 (72)	57 (46)	
Moderate	52 (17)	52 (13)	16 (13)	< 0.01
Fluent	123 (41)	56 (14)	51 (41)	
Total time in clinic (months)—median [IQR^d^]	15.4 [6–25]	17.2 [9–27]	16.6 [8–26]	0.20
Number of medical diagnoses^e^—median [IQR^d^]	4 [2–7]	7 [4–11]	4 [3–9]	< 0.01

^a^Privately sponsored refugee

^b^Government assisted refugee

^c^Refugee claimant

^d^Interquartile range

^e^Medical diagnoses at time of data extraction

^f^Results were analyzed with age at last appointment

The study was reviewed and approved by the University of Calgary Research Ethics Board (REB15-3264).

## Results

### Participants & Descriptive Data

We included a total of 812 refugee patients in our analysis, with 299 PSRs, 389 GARs and 124 claimants. The median age of PSRs was 34.0 [IQR: 29–41] years, GARs 33.0 [IQR: 28–42] years, and claimants 35.0 [IQR: 29–46] years. Overall, study participants represented 57 countries of origin. The top five countries of origin were Eritrea (21%), Syria (12%), Ethiopia (12%), Iraq (11%) and Somalia (6%), with 49% of African origin. Most were married (71%) and had limited English proficiency (57%). PSRs spent a median of 15.4 [IQR: 6–25] months in clinic and had a median of 4 [IQR: 2–7] medical diagnoses; GARs spent a median of 17.2 [IQR: 9–27] months in clinic and had a median of 7 [IQR: 4–11] medical diagnoses; and claimants spent a median of 16.6 [IQR: 8–26] months in clinic and had a median of 4 [IQR: 3–9] diagnoses.

### Main Results

Overall, 718/812 eligible patients (88%) were offered CCS and 629/812 (77%) completed CCS (Table 2). Among all patients offered CCS screening, 89 (12%) did not complete CCS. While claimants were offered screening less frequently (80%) than PSRs (91%) or GARs (89%) (P < 0.01), there was no significant difference in CCS completion between refugee categories.

**Table 2:** Eligible women who were offered and received cervical cancer screening by refugee category at the Mosaic Refugee Health Clinic, 2011–2016 (N = 812)

	PSR^a^ n = 299	GAR^b^ n = 389	RC^c^ n = 124	P value
Pap test offered—n (%)	272 (91)	347 (89)	99 (80)	< 0.01
Pap test completed—n (%)	241 (81)	296 (76)	92 (74)	0.22

^a^Privately sponsored refugee

^b^Government assisted refugee

^c^Refugee claimant

### Primary Analyses

**Fig. 2:**
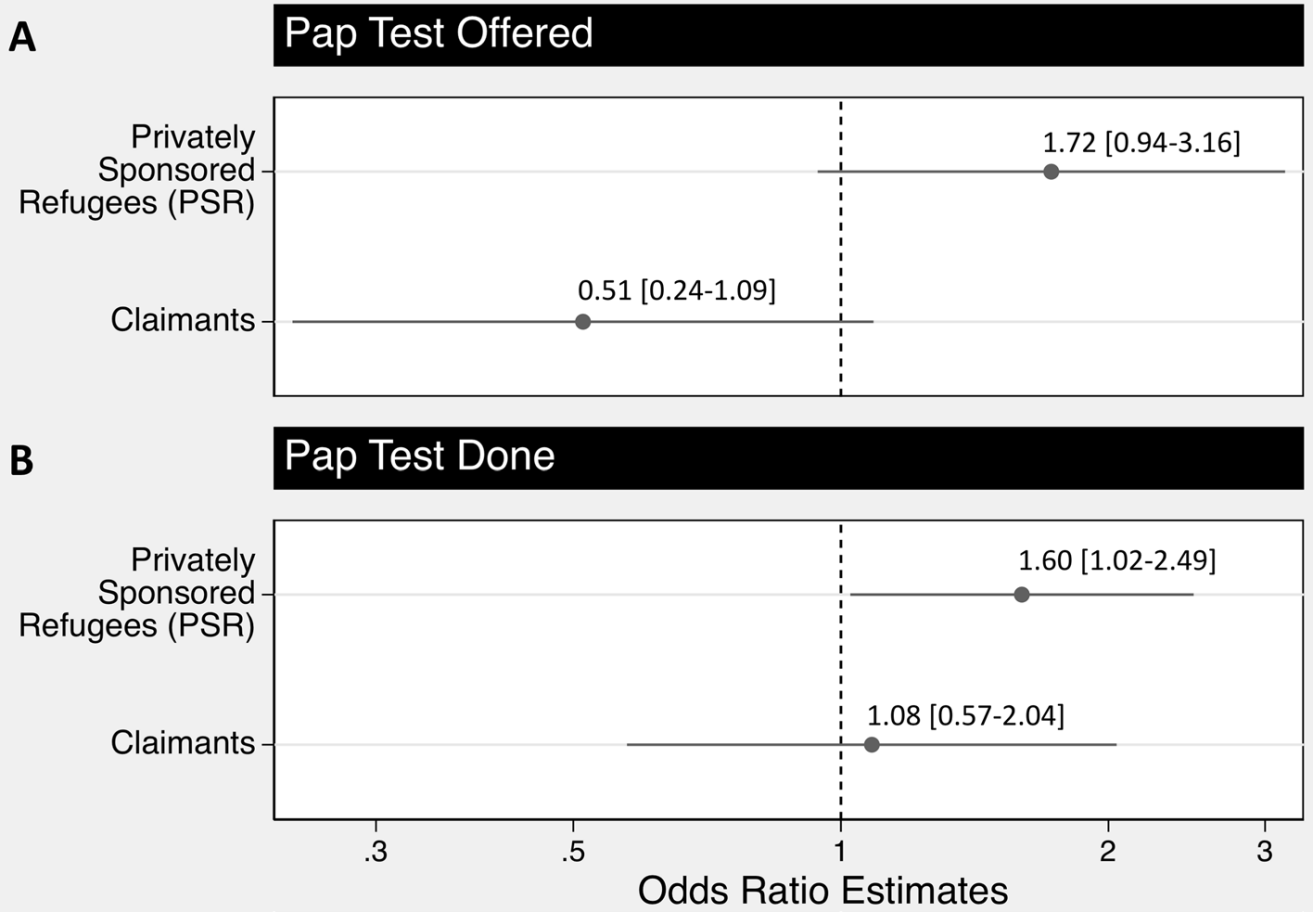
Adjusted multivariable regression analysis of cervical cancer screening test offers (**A**) and completion (**B**) by refugee category, compared to government assisted refugees at the Mosaic Refugee Health Clinic, 2011–2016 (N = 812)

After adjusting for sociodemographic and clinical factors we found that, when compared to GARs, only PSRs had a higher odds of completing a CCS test (P < 0.04) (Fig. 2). In contrast, after adjusting for sociodemographic and clinical factors, we found no significant differences in the odds of CCS test offers among PSRs or claimants, when compared to GARs.

### Other Factors

Our multivariable regression analysis also revealed that patients at age extremes had decreased odds of having CCS offered (21–24 years and 65–71 years) (Table 3). Refugees aged 65–71 also had decreased odds of completing CCS. Patients who identified as single had decreased odds of being offered and completing CCS screening. Region of origin and English proficiency were not associated with test offers or completion; however the odds of both test offers and completion increased with increased number of diagnoses and months in clinic.

**Table 3:** Adjusted odds ratios (with 95% confidence intervals) for cervical cancer screening tests offered and completed, with respect to sociodemographic and clinical variables at the Mosaic Refugee Health Clinic, 2011–2016 (N = 812)

Variables	Pap test offered	Pap test completed
	Adjusted odds ratio [95% CI^a^]	Adjusted odds ratio [95% CI^a^]
Refugee category	Calculated vs. GAR^b^	Calculated vs. GAR^b^
PSR^c^	1.73 [0.94–3.16]	1.60 [1.02–2.49]^*^
RC^d^	0.51 [0.24–1.09]	1.08 [0.57–2.04]
Age bands	Calculated vs. 25–64	Calculated vs. 25–64
21–24	0.40 [0.22–0.72]**	0.62 [0.37–1.04]
65–71	0.05 [0.01–0.27]***	0.10 [0.03–0.38]*
Region of origin	Calculated vs. Africa	Calculated vs. Africa
Americas	1.61 [0.50–5.20]	1.43 [0.53–3.87]
Asia	0.91 [0.53–1.56]	0.96 [0.64–1.45]
Europe	0.78 [0.23–2.63]	0.55 [0.19–1.57]
Marital status	Calculated vs. Married	Calculated vs. Married
Separated/Divorced	0.65 [0.24–1.74]	0.61 [0.30–1.22]
Single	0.36 [0.20–0.65]**	0.55 [0.34–0.89]*
Unknown	0.75 [0.11–4.85]	0.55 [0.11–2.71]
Widowed	1.48 [0.36–6.16]	1.57 [0.61–4.10]
English fluency	Calculated vs. No English	Calculated vs. No English
Some English	0.76 [0.39–1.48]	1.06 [0.62–1.81]
Fluent English	1.28 [0.69–2.37]	1.16 [0.73–1.82]
Total time in clinic (months)	1.03 [1.01–1.06]*	1.05 [1.03–1.06]***
Number of medical diagnoses	1.18 [1.08–1.28]***	1.07 [1.02–1.12]**

^a^Confidence Interval

^b^Privately sponsored refugee

^c^Government assisted refugee

^d^Refugee claimant

*P < 0.05

**P < 0.01

***P < 0.001

### Exploratory Analysis—CCS outcomes

In our exploratory analysis of CCS pathology results we did not identify any cervical cancer cases. Pathology results were available for 603 patients and of this subset, 570 (95%) were negative, 6 (1%) showed low-grade squamous intraepithelial lesions, 5 (0.8%) were high-grade squamous intraepithelial lesions, 12 (1.9%) were atypical cells of undetermined significance and 11 (1.8%) were not reported or processed (lab or collection issue).

## Discussion

### Key Results

The objective of our study was to determine how many eligible patients were offered, and subsequently received CCS, and whether this was impacted by refugee category during a time of cuts to health care coverage for refugees. We found that most eligible refugee patients were routinely both offered (88%) and received (77%) CCS at a dedicated interdisciplinary refugee primary care clinic. Unadjusted results suggested that claimants were offered screening less frequently than PSRs or GARs. However, after adjusting for factors known to influence CCS rates, the analysis revealed no significant difference in screening test offers between refugee categories. With regards to completion rates, PSRs had increased odds (1.6, 95% CI 1.02–2.49) of completing CCS compared with GARs, with no difference for claimants. No cases of cervical cancer were identified in our study, however those with low- or high-grade findings would have been flagged for further follow up appropriate to their result. These findings suggest that despite uncertainty for both patients and providers due to limitations to the IFHP, refugee claimants still had access to effective CCS at a dedicated refugee clinic.

Our results also suggest that a dedicated interdisciplinary refugee clinic achieves high CCS rates, with a seemingly higher CCS completion rate higher than both the Alberta provincial average from 2011 to 2016 (67%) as well as WHO’s 70% screening target for all women by 2030 [5, 34, 35]. High CCS rates have also been reported at specialized refugee clinics in Toronto and Philadelphia [16, 27, 36, 37], suggesting that despite known barriers to care for refugees, dedicated interdisciplinary refugee clinics can provide effective screening in a traditionally underserved population [6].

### Interpretation

While not examined in the context of CCS, previous studies suggest that PSRs integrate into the Canadian healthcare system more successfully than GARs, with better language fluency, employment possibilities and health status [38]. Clinically, MRHC physicians observe PSRs attending appointments with their sponsors who often help with system navigation and who discuss and support screening maneuvers. The additional support provided to PSRs, as well as generally higher education and language proficiency in French and English [39], may explain their increased likelihood of completing CCS in our study [13–15]. As expected, patients who had more diagnoses or who were seen over a longer period were more likely to be screened. Additionally, while many studies have identified low English proficiency as a barrier to CCS among immigrants and refugees, English language proficiency did not impact rates of CCS offered or completed in our study [14, 40]. This suggests that a dedicated refugee clinic that routinely uses professional telephone-based translation services can overcome this significant barrier [41, 42].

While not our primary focus, we found other patient factors were associated with our CCS outcomes of interest. Patients identified as single were at lower odds of having a test offered and completed. This is in keeping with other studies in immigrants where being married was positively associated with CCS [43–46]. Also, our study covered a large patient age range, including childbearing and menopause life stages. We found that CCS tests were offered less frequently to younger and older patients, completed less frequently for those who were older, and completed with similar frequency for those between ages 25–65. While this possibly reflects a trend to delay starting CCS, studies in Ontario have similarly found that in immigrants to Canada, and in the general population, age at the extremes of screening guidelines is associated with decreased likelihood to complete CCS [10, 15]. Although current guidelines generally support this decrease in screening for those over 69 years, some question whether these assumptions and upper age limits should be revisited as the morbidity from cervical cancer in older age groups remains significant [40].

### Limitations

Our study must be assessed within the context of its limitations. First, we did not assess several factors known to impact CCS among immigrants and refugees as these data were unavailable for this analysis. These factors include provider gender, a patients’ parity and level of education. Second, while this is a single site study with limited sample size, to our knowledge this study presents the largest cohort analysis of CCS in newly arrived refugees in Canada and includes several other valuable demographic and clinical factors. Third, because intimate exams were routinely performed only on follow up visits, only patients who attended two appointments were included; thus, we may have overestimated the CCS rate among patients less likely to attend more than once, including claimants. Fourth, despite having embedded gynecology specialists within the refugee clinic whose test results would be captured in the EMR, patients may have completed screening at other clinics if they were seen by an external specialist or went to another provider, leading to lower observed completion rates. Finally, the generalizability of our findings may be limited as our study was conducted at a dedicated refugee clinic, however, effective screening rates in this setting lend credence to a growing body of literature suggesting that health needs for underserved communities are well managed in dedicated clinics [47].

## New Contribution to the Literature

This study provides a unique analysis of offered and completed CCS among newly arrived refugees, and examines the association of refugee category on these outcomes. Eligible patients in all refugee categories were offered and completed CCS at high rates despite restrictions to the Interim Federal Health Program for claimants during the study period. Between refugee categories, PSRs were more likely than GARs to complete screening. These findings show that despite a period of uncertainty for providers and patients, which could have negatively impacted care and screening rates, a dedicated refugee clinic can provide high rates of CCS to marginalized refugee patients.

## Conclusion

Refugees face multiple barriers to care and are at risk of inadequate CCS. Despite this, we found that patients at a specialized interdisciplinary refugee health clinic were offered and completed CCS at high rates, suggesting this model may improve outcomes and health equity in this population. Future research should examine the likelihood of refugees receiving subsequent and repeat CCS tests, both in follow up to abnormal findings, as well as for ongoing routine screening after transitioning to community primary care clinics.
